# Associations between the Nutrient Profiling System Underlying the Nutri-Score Nutrition Label and Biomarkers of Chronic Low-Grade Inflammation: A Cross-Sectional Analysis of a Middle- to Older-Aged Population

**DOI:** 10.3390/nu14153122

**Published:** 2022-07-29

**Authors:** Seán R. Millar, Pilar Navarro, Janas M. Harrington, Ivan J. Perry, Catherine M. Phillips

**Affiliations:** 1 HRB Centre for Health and Diet Research, School of Public Health, University College Cork, T12 XF62 Cork, Ireland; j.harrington@ucc.ie (J.M.H.); i.perry@ucc.ie (I.J.P.); catherine.phillips@ucd.ie (C.M.P.); 2 School of Public Health, Physiotherapy and Sports Science, University College Dublin, D04 V1W8 Dublin 4, Ireland; mpilar_ns@hotmail.com

**Keywords:** nutri-score, diet, chronic, inflammation, biomarkers

## Abstract

Low-grade systemic inflammation is associated with a range of conditions. Diet may modulate inflammation and public health strategies are needed to guide consumers’ dietary choices and help prevent diet-related disease. The Food Standards Agency nutrient profiling system (FSAm-NPS) constitutes the basis of the five-colour front-of-pack Nutri-Score labelling system. No study to date has examined FSAm-NPS dietary index associations with biomarkers of inflammation. Therefore, our objective was to test relationships between the FSAm-NPS and a range of inflammatory biomarkers in a cross-sectional sample of 2006 men and women aged 46–73 years. Individual participant FSAm-NPS scores were derived from food frequency questionnaires. Pro-inflammatory cytokine, adipocytokine, acute-phase response protein, coagulation factor and white blood cell count concentrations were determined. Correlation and linear regression analyses were used to examine FSAm-NPS relationships with biomarker levels. In crude and adjusted analyses, higher FSAm-NPS scores, reflecting poorer nutritional quality, were consistently and positively associated with biomarkers. In fully adjusted models, significant associations with concentrations of complement component 3, c-reactive protein, interleukin 6, tumour necrosis factor alpha, resistin, white blood cell count, neutrophils, eosinophils and the neutrophil-to-lymphocyte ratio persisted. These results suggest that dietary quality, determined by Nutri-Score rating, is associated with inflammatory biomarkers related to health.

## 1. Introduction

Low-grade systemic inflammation is considered to be a condition related to many chronic diseases including type 2 diabetes, cardiovascular disease, neurodegenerative disease and many cancers [1,2,3,4,5,6]. Habitual dietary intake is thought to modulate inflammation through complex interactions between foods and nutrients with bioactive properties [7,8] and is a potential therapeutic target to reduce metabolic dysfunction and risk of chronic disease development [7,9,10,11,12]. Research has shown that more fruits and vegetables, dietary fibres and less sugars and saturated fats should be consumed for better health. However, studies have also suggested that it is important to characterise dietary quality as a whole. Importantly, this allows combinations of nutrients and other compounds that act synergistically within a diet to be captured [13].

As foods and beverages with low nutritional and high caloric content are believed to be drivers of diet-related conditions [14,15], front-of-pack labelling may be an important public health policy measure that could be instigated to promote healthy eating and prevent disease [16,17]. The Nutri-Score labelling system classifies foods and beverages into five colour-coded categories, from category A (which indicates better nutritional quality) to category E (which indicates poorer nutritional quality). This is assessed using the Food Standards Agency nutrient profiling system (FSAm-NPS), which is a revised version of a profiling system created by the British Food Standards Agency [18,19,20]. The FSAm-NPS was developed to prevent nutrition-related conditions by assigning a score to foods or beverages per 100 g content of energy, sugars, sodium, protein, saturated fatty acids, fruits, vegetables, dietary fibre, legumes and nuts [21].

It is important to test the validity of the FSAm-NPS scoring system by examining relationships between the nutritional quality of foods graded by the FSAm-NPS and health outcomes [18]. Accordingly, research has demonstrated that consumption of food products with lower FSAm-NPS scores (representing higher nutritional quality) is related to more favourable outcomes for weight gain, metabolic syndrome, cardiovascular disease, cancer and mortality [21]. However, it is also important to examine relationships between the FSAm-NPS and biomarkers of disease risk, as a dietary scoring system’s validity is also dependent on how it can discriminate between subjects on applicable intermediate markers of health [22,23]. Although studies have demonstrated associations between dietary quality and circulating biomarkers of inflammation [7], only a limited number of inflammatory biomarkers have been investigated in this context [24]. In addition, no study has assessed relationships between the dietary profile of foods underlying the Nutri-Score nutrition label and biomarkers of chronic low-grade inflammation.

Therefore, in this study we examined associations between the FSAm-NPS scoring system and a large range of biomarkers of systemic inflammation and raised immune activation, using a random sample of 2006 men and women aged 46–73 years, to test the hypothesis that nutritional quality according to Nutri-Score rating would be associated with circulating levels of inflammatory biomarkers.

## 2. Materials and Methods

### 2.1. Study Population and Setting

The Mitchelstown cohort study (Phase II of the Cork and Kerry Diabetes and Heart Disease Study) was conducted between 2010 and 2011 in order to provide an updated profile of the glucose tolerance status, cardiovascular health and their related factors in an Irish middle- to older-aged population sample. A representative sample was recruited from the Livinghealth clinic, a large primary care centre in Mitchelstown, County Cork, Ireland. We used stratified random sampling to recruit equal numbers of men and women from all registered attending patients in the 45–70-year age group. After excluding duplicates, deaths and individuals who were incapable of consenting or who did not attend appointments, 3051 were invited to participate in the study. Of these subjects, about two-thirds (2047, 49% male) participated. Dietary data were available for 2006 subjects. Details of the study methodology have been reported previously [25].

### 2.2. Laboratory Procedures

After an overnight fast, study participants attended the clinic in the morning and blood samples were taken on arrival. Complement component 3 (C3) levels were assessed by immunoturbidimetric assay (RX Daytona; Randox Laboratories) and c-reactive protein (CRP), tumour necrosis factor-alpha (TNF-α), interleukin 6 (IL-6), adiponectin, leptin, resistin and plasminogen activator inhibitor-1 (PAI-1) levels were measured using a biochip array system (Evidence Investigator; Randox Laboratories, Ardmore, UK). White blood cell count (WBC), neutrophil, lymphocyte, monocyte, eosinophil and basophil concentrations were assessed by flow cytometry technology at the Cork University Hospital Biochemistry Laboratory. The neutrophil-to-lymphocyte ratio (NLR) was calculated as neutrophils divided by lymphocytes. Glucose concentrations were determined using a glucose hexokinase assay (Olympus Life and Material Science Europa Ltd., Lismeehan, Co. Clare, Ireland) and HbA1c levels were measured on an automated high-pressure liquid chromatography instrument Tosoh G7 [Tosoh HLC-723 (G7), Tosoh Europe N.V, Tessenderlo, Belgium].

### 2.3. Data Collection

A self-completed questionnaire was also used to collect data. These data included information on age, sex, education, prescription anti-inflammatory medication use, smoking status and presence of type 2 diabetes. The validated International Physical Activity Questionnaire (IPAQ) [26] was utilised to assess physical activity levels. Anthropometric measurements were recorded by trained researchers according to a standardised protocol using calibrated instruments. Height was measured with a portable Seca Leicester height/length stadiometer (Seca, Birmingham, UK) and weight was measured using a portable electronic Tanita WB-100MA weighing scale (Tanita Corp, Arlington Heights, IL, USA). Body mass index (BMI) was calculated using weight and height measurements.

### 2.4. Dietary Intake Assessment

Diet was evaluated using the self-completed European Prospective Investigation into Cancer and Nutrition (EPIC) Food Frequency Questionnaire (FFQ) [27]. This FFQ was modified to reflect the Irish diet by the National Nutrition Surveillance Centre in Ireland and has been validated for use in the Irish population [28,29]. Data on food frequency consumption during the past 12 months were gathered. Using a tailored computer programme (FFQ Software Version 1.0; developed by the National Nutrition Surveillance Centre, School of Public Health, Physiotherapy and Sports Science, University College Dublin, Belfield, Dublin 4, Ireland) the intake of energy and nutrients was computed from the FFQ data by linking food equivalents in McCance and Widdowson Food Tables to food frequency selections [30].

### 2.5. FSAm-NPS Dietary Index Computation

As described previously [19,20], the FSAm-NPS is a modified version of the original FSA-NPS developed in the United Kingdom and categorises foods and drinks as ‘healthier’ and ‘less healthy’. Importantly, the FSAm-NPS incorporated adaptations in the FSA-NPS cut-offs to allow for more than two categories reflecting nutritional quality. After recommendations from the French National Nutrition and Health Program and the French High Council for Public Health, additional modifications included scoring criteria for cheese, added fats and beverages [31]. The FSAm-NPS score, which punctuates the amount of nutrients per 100 g of product, was calculated for all foods and beverages in the Mitchelstown FFQ as follows: points (0–10) are allocated for total sugars (g), saturated fatty acids (g), sodium (mg), and energy (kJ) (i.e., nutrients that should be consumed in limited amounts) and can be balanced by opposite points (0–5) allocated for dietary fibres (g), proteins (g), and fruits/vegetables/legumes/nuts (percent) (i.e., nutrients/components that should be promoted). The final FSAm-NPS score was therefore on a discrete continuous scale ranging from −15 (most healthy) to +40 (least healthy). 

To obtain a dietary score at the individual level, the FSAm-NPS was computed as an energy weighted mean of the FSAm-NPS scores of all foods and beverages consumed by a participant using the following equation (FSi represents the score of food/beverage *i*, *E_i_* the energy intake from food/beverage *i*, and n the total number of food/beverage consumed):

$$\mathrm{FSAm}-\mathrm{NPS}\;\mathrm{dietary}\;\mathrm{index}=\frac{\sum_{i=1}^{n}\left(FS_{i}\;E_{i}\right)}{\sum_{i=1}^{n}E_{i}}$$

Higher FSAm-NPS dietary index scores reflect lower nutritional quality in the total foods consumed. More details on the FSAm-NPS scoring system can be found in Supplementary File S1 and previous publications [32,33,34,35,36,37].

### 2.6. Classifcation and Scoring of Variables

Educational levels recorded in the self-completed questionnaire included ‘some primary (not complete)’, ‘primary or equivalent’, ‘intermediate/group certificate or equivalent’, ‘leaving certificate or equivalent’, ‘diploma/certificate’, ‘primary university degree’ and ‘postgraduate/higher degree’. We recoded these into a binary variable: ‘primary education only’ (finished full-time education at 13 years or younger) and ‘intermediate or higher’. Smoking status was defined as never, former and current smoker and was recoded as: ‘never/former smoker’ or ‘current smoker’. Physical activity was categorised as low, moderate and high levels of activity using the IPAQ and was then recoded as: ‘moderate/high’ or ‘low’ level physical activity. Type 2 diabetes was determined by a self-reported physician diagnosis or as a fasting glucose level ≥7.0 mmol/L or an HbA1c level ≥6.5% (≥48 mmol/mol) [38].

### 2.7. Statistical Analysis

Descriptive characteristics were examined according to FSAm-NPS dietary index quartiles. In our analyses we show categorical variables as percentages and continuous variables as a mean (plus or minus one standard deviation) or a median and interquartile range for skewed data. Trend relationships were determined using a Jonckheere test, a linear-by-linear chi-square or an ANOVA. We used Spearman’s rank-order test to examine correlative strengths between FSAm-NPS scores and biomarker concentrations.

Skewed biomarker data were log-transformed and FSAm-NPS scores were standardised for linear regression analyses which examined associations between the FSAm-NPS and biomarker levels. Four models were run to test relationships. The final model was adjusted for age, sex, anti-inflammatory medication use, physical activity, education, smoking, type 2 diabetes and BMI. Multivariate models were not adjusted for energy intake as this is taken into account in the FSAm-NPS dietary index scoring system.

Data analysis was conducted using Stata SE Version 13 (Stata Corporation, College Station, TX, USA) for Windows. For all analyses, a *p* value (two-tailed) of less than 0.05 was considered to indicate statistical significance.

## 3. Results

### 3.1. Descriptive Characteristics

Table 1 shows characteristics of the study population according to FSAm-NPS dietary index quartiles. Higher scores indicate lower nutritional quality/poorer diet whereas lower scores indicate a healthier diet. Subjects with a higher score (quartile 4 compared to quartile 1) were more likely to be male, to have lower levels of physical activity, were less likely to report anti-inflammatory medication use and had higher (lower for adiponectin) concentrations of inflammatory and thrombotic biomarkers than did those who consumed higher quality diets. 

Daily energy intake and dietary macronutrient composition were noticeably different across FSAm-NPS dietary index quartiles (Table 2). Participants with lower FSAm-NPS scores (corresponding to a more favourable Nutri-Score rating) demonstrated lower consumption of saturated fatty acids, polyunsaturated fatty acids, monounsaturated fatty acids, carbohydrates and a greater consumption of fibre. Examination of daily number of servings based on food pyramid recommendations revealed that those with a more favourable FSAm-NPS score consumed greater amounts of fruits and vegetables and lesser amounts of high fat/sugar foods and drink products.

### 3.2. Correlation Analysis

In correlation analyses (Table 3), weak but significant positive correlations between the FSAm-NPS dietary index and biomarkers were observed for concentrations of C3, CRP, IL-6, TNF-α, WBC, neutrophils, the NLR, monocytes and eosinophils. Adiponectin levels were inversely correlated with the FSAm-NPS index.

### 3.3. Linear Regression

Table 4 shows linear regression models demonstrating relationships between standardised FSAm-NPS scores and biomarkers. In crude and adjusted analyses, consistent and positive associations were observed between the FSAm-NPS index and inflammatory and thrombotic markers. In fully adjusted models, significant associations with concentrations of C3 (β = 1.767, *p* = 0.002), CRP (β = 0.047, *p* = 0.004), IL-6 (β = 0.042, *p* = 0.016), TNF-α (β = 0.023, *p* = 0.009), resistin (β = 0.029, *p* = 0.005), WBC (β = 0.016, *p* = 0.01), neutrophils (β = 0.024, *p* = 0.002), eosinophils (β = 0.037, *p* = 0.015) and the NLR (β = 0.021, *p* = 0.028) persisted.

## 4. Discussion

In this study of 2006 middle- to older-aged men and women we examined relationships between the dietary profile of foods underlying the Nutri-Score nutrition label and a range of pro-inflammatory cytokines, adipocytokines, acute-phase response proteins, coagulation factors and white blood cells. We report significant positive associations between higher FSAm-NPS dietary index scores, reflecting poorer dietary quality, and concentrations of C3, CRP, IL-6, TNF-α, resistin, WBC, neutrophils, eosinophils and the NLR in analyses which adjusted for a range of potential confounders. As other studies have shown, our findings also suggest systemic inflammation as a biological mechanism linking dietary quality with health effects [7,39]. 

Chronic low-grade inflammation is thought to contribute to the development chronic conditions and evidence suggests that nutrients and food components modulate inflammatory status. Vitamins C and E, selenium and carotenoids are antioxidants and these may reduce development of reactive species that instigate disease development through inflammation [40]. It is believed that fruits and vegetables that contain these nutrients and others provide anti-inflammatory benefits while studies indicate that excessive amounts of red and processed meats, sugar-sweetened beverages and refined grains are pro-inflammatory through a variety of mechanisms [41,42]. Considering this, studies have emphasised the need to test the relationship between diet and systemic inflammation by examining dietary patterns [7]. Dietary scoring systems reflect the fact that foods are eaten in combination and this removes the limitation that assessment of single nutrients may not reflect the overall quality of diet or take into account interactions among nutrients [43]. 

This concept has been supported in findings from a number of studies. A meta-analysis which examined the Dietary Inflammatory Index (DII^®^), a dietary score created to capture the inflammatory potential of diet based on dietary components, revealed that individuals with more pro-inflammatory diets (i.e., the highest DII score), had a 36% increased risk of cardiovascular disease incidence and mortality relative to those with the lowest DII score [44]. Fung et al. reported that the Dietary Approaches to Stop Hypertension diet had favourable effects in reducing inflammation in 24-year follow-up of women from the Nurses’ Health Study [45] while Richard et al. found that consuming the Mediterranean Diet significantly reduced inflammation; this effect was noted even in the absence of weight loss [46]. A recent literature review also identified an association between higher quality diet and more favourable inflammatory biomarker levels [24]. 

We are unaware of any research that has explored FSAm-NPS dietary index relationships with biomarkers of systemic inflammation. Nevertheless, several studies have demonstrated associations between Nutri-Score rating and risk of chronic conditions and all-cause mortality. The French SU.VI.MAX study which followed 6435 subjects over 13 years found that consumption of foods with lower FSAm-NPS scores was associated with a lower risk of developing chronic diseases, including cancers, metabolic syndrome and cardiovascular disease [33,34,35]. Data from the SUN cohort (20,503 subjects; 10-year follow-up) [15] and the ENRICA cohort (12,054 subjects; 10-year follow-up) [47] in Spain revealed that consuming foods with a poorer Nutri-Score classification was associated with a higher rate of cancer mortality, cardiovascular disease mortality and all-cause mortality. 

In the present study, we found the FSAm-NPS dietary index to be associated with eight of the 14 examined inflammatory/thrombotic biomarkers and the NLR in multivariate analyses. Consequently, our results might suggest systemic inflammation as a mechanism underlying observed associations between Nutri-Score rating and morbidity and mortality. It should be noted, however, that relationships between FSAm-NPS scores and biomarkers of chronic low-grade inflammation and raised immune activation were modest in our sample; examination of other biomarker associations with the FSAm-NPS could provide further mechanistic insights. In addition, it is also important to note that age-related changes in metabolic risk factors occur. Therefore, future studies should examine FSAm-NPS-biomarker associations across the life course [39]. 

Unlike other dietary scores, components of the FSAm-NPS index cannot be studied separately. This is because the FSAm-NPS is first calculated at the food level and then aggregated at the individual level [36]. Nevertheless, studies have indicated that subjects with a dietary intake corresponding to a better FSAm-NPS score have a lower consumption of sweet and fatty snacking products, lower saturated fatty acid intakes and higher consumption of fruits and vegetables [48]. These findings were also observed in our research, suggesting that eating foods that are better ranked on the Nutri-Score scale is associated with better overall dietary nutritional quality [18]. In addition, a study by De Temmerman et al. [49], which investigated the impact of the Nutri-Score and its five categories on consumers’ perceived perceptions of healthiness and purchasing intentions, found that the presence of the Nutri-Score enabled respondents to better assess the healthiness of food and beverage products. Surveys carried out in France, Spain, Belgium and Germany have additionally shown that the Nutri-Score is perceived favourably by consumers. These surveys also found that the Nutri-Score is the preferred format when compared to other nutrition labels tested; this was found to be particularly true in populations with the lowest level of nutritional knowledge [18]. It should be noted that controversy exists regarding the Nutri-Score and its proposed adoption by the European Union Commission as a front-of-pack labelling system for European Union member states. However, findings from our research and other studies suggest that the Nutri-Score labelling system may be an effective tool to help consumers make more healthy food choices and also prevent inflammatory dysregulation and inflammatory-related disease.

This study has several strengths. This research is the first to examine relationships between the dietary profile of foods underlying the Nutri-Score nutrition label and a wide range of markers of chronic low-grade inflammation and raised immune activation in a middle- to older-aged population. With an ageing world population [50], it is likely that the number of patients with non-communicable diseases will increase. Front-of-pack labelling tools that guide consumers into adopting a healthier diet might help prevent against systemic inflammation and related conditions; this may be of particular relevance to older adults. Other strengths of our study include the relatively large number of middle- to older-aged study participants with regard to the biomarkers examined, equal representation by sex and the use of validated questionnaires to collect data. 

Despite these strengths, there are a number of limitations. As this is a cross-sectional study, our findings preclude drawing conclusions regarding the temporal direction of relationships; this limits inference with respect to causality. The use of self-reported questionnaires is subject to potential inaccuracies [51,52,53] and the generalisability of our findings may also be limited. As these data were collected from a single primary care-based sample, they may not be representative of the general population. However, Ireland represents a generally ethnically homogeneous population [54]. It has also been noted in previous research that approximately 98% of Irish adults are registered with a GP. Consequently, even in the absence of a universal patient registration system, it is believed to be possible to perform population-based epidemiological studies that are representative using our methods [55]. 

## 5. Conclusions

In conclusion, findings from this research demonstrate that higher FSAm-NPS scores, reflecting poorer dietary quality, are associated with a more pro-inflammatory profile in middle- to older-aged adults. More favourable inflammatory status may be a potential mechanism linking higher quality diet and reported health benefits of a healthy diet according to Nutri-Score rating. The Nutri-Score labelling system may be an important tool to help consumers make healthy food choices, achieve better dietary quality/nutritional status and also help prevent inflammatory dysregulation and inflammatory-related disease. Further examination of other biomarker associations with the FSAm-NPS dietary index could provide additional mechanistic insights into the relationship between diet and disease.

## Figures and Tables

**Table 1:** Descriptive characteristics and inflammatory profiles of the study population according to FSAm-NPS dietary index quartiles.

Variable	FSAm-NPS Dietary Index Quartiles (n = 2006)				
	Q1	Q2	Q3	Q4	*p _trend_*
Age (median)	59.6 (55.0–64.0)	59.2 (54.0–63.5)	59.0 (54.0–63.5)	59.0 (54.0–64.0)	0.053
Male (%)	198 (39.4)	221 (44.1)	261 (52.1)	303 (60.4)	<0.001
Primary education only (%)	126 (26.9)	131 (27.6)	122 (26.1)	148 (31.2)	0.219
On anti-inflammatory medications (%)	96 (19.5)	86 (17.6)	86 (17.6)	67 (13.6)	0.019
Type 2 diabetes (%)	53 (10.6)	44 (8.8)	44 (8.8)	39 (7.8)	0.143
Current smoker (%)	63 (12.8)	64 (13.0)	85 (17.1)	77 (15.4)	0.091
Low-level physical activity (%)	191 (40.4)	225 (46.1)	244 (51.6)	257 (55.4)	<0.001
BMI, kg/m^2^ (mean)	28.3 ± 4.6	28.7 ± 4.8	28.7 ± 4.7	28.6 ± 4.8	0.329
C3, mg/dL (mean)	133.43 ± 25.4	136.53 ± 23.5	137.18 ± 24.5	136.20 ± 25.2	0.073
CRP, ng/mL (median)	1.26 (0.93–2.13)	1.36 (0.98–2.32)	1.38 (0.98–2.33)	1.41 (0.99–2.36)	0.01
IL-6, pg/mL (median)	1.61 (1.14–2.72)	1.77 (1.17–2.85)	1.82 (1.18–2.95)	1.96 (1.30–3.09)	<0.001
TNF-α, pg/mL (median)	4.78 (4.74–7.22)	5.91 (4.80–7.29)	6.03 (4.95–7.32)	6.22 (5.11–7.36)	0.003
Adiponectin, ng/mL (median)	5.42 (3.15–8.31)	4.83 (3.00–7.70)	4.73 (2.93–7.50)	4.00 (2.73–6.63)	<0.001
Leptin, ng/mL (median)	2.00 (1.12–3.21)	2.00 (1.03–3.35)	1.98 (1.19–3.31)	1.71 (1.00–2.83)	0.46
Resistin, ng/mL (median)	5.06 (3.90–6.66)	5.00 (3.95–6.65)	4.92 (3.90–6.70)	5.20 (3.92–6.92)	0.377
PAI-1, ng/mL (mean)	27.17 ± 13.8	26.66 ± 11.2	27.87 ± 12.5	27.98 ± 12.6	0.153
WBC, 10^9^/L (median)	5.40 (4.70–6.50)	5.70 (4.70–6.70)	5.70 (4.90–7.00)	5.90 (5.00–7.00)	<0.001
Neutrophils, 10⁹/L (median)	2.97 (2.39–3.76)	3.09 (2.46–3.87)	3.18 (2.53–4.00)	3.26 (2.70–4.17)	<0.001
Lymphocytes, 10⁹/L (median)	1.73 (1.42–2.12)	1.74 (1.44–2.15)	1.76 (1.41–2.14)	1.75 (1.43–2.17)	0.32
NLR (median)	1.73 (1.32–2.24)	1.74 (1.36–2.23)	1.76 (1.41–2.31)	1.86 (1.48–2.38)	<0.001
Monocytes, 10⁹/L (median)	0.48 (0.38–0.59)	0.49 (0.39–0.60)	0.50 (0.41–0.63)	0.52 (0.43–0.65)	<0.001
Eosinophils, 10⁹/L (median)	0.16 (0.11–0.25)	0.17 (0.10–0.25)	0.18 (0.12–0.27)	0.18 (0.12–0.27)	0.005
Basophils, 10⁹/L (median)	0.031 (0.02–0.04)	0.032 (0.02–0.04)	0.033 (0.02–0.04)	0.033 (0.02–0.04)	0.129

Abbreviations: BMI: body mass index; C3: complement component 3; CRP: c-reactive protein; FSAm-NPS: Food Standards Agency nutrient profiling system; IL-6: interleukin 6; TNF-α: tumour necrosis factor alpha; PAI-1: plasminogen activator inhibitor 1; WBC: white blood cell count; NLR: neutrophil-to-lymphocyte ratio. *p* for trend determined from a Jonckheere test, a linear-by-linear chi-square or an ANOVA.

**Table 2:** Nutritional intake of the study population according to FSAm-NPS dietary index quartiles.

Variable	FSAm-NPS Dietary Index Quartiles (n = 2006)				
	Q1	Q2	Q3	Q4	*p _trend_*
**Dietary composition**					
Energy intake, kcal (mean)	1808.8 ± 740.0	1975.4 ± 752.7	2133.7 ± 835.7	2211.3 ± 874.4	<0.001
Fat, g/d (mean)	60.1 ± 29.6	72.5 ± 32.7	82.5 ± 37.5	93.8 ± 42.4	<0.001
SFA, g/d (mean)	18.1 ± 8.7	23.3 ± 11.1	28.6 ± 13.0	38.0 ± 17.1	<0.001
PUFA, g/d (mean)	13.6 ± 9.4	15.5 ± 8.4	16.7 ± 9.7	16.1 ± 9.7	<0.001
MUFA, g/d (mean)	19.3 ± 9.9	23.2 ± 10.8	26.1 ± 12.3	28.6 ± 13.3	<0.001
Carbohydrate, g/d (mean)	232.5 ± 106.9	246.1 ± 104.5	259.3 ± 112.0	260.2 ± 115.7	<0.001
Protein, g/d (mean)	90.6 ± 41.0	90.8 ± 33.1	95.1 ± 37.3	90.6 ± 38.8	0.556
Sugar, g/d (mean)	101.7 ± 64.8	105.3 ± 56.1	106.4 ± 53.4	108.1 ± 61.8	0.086
Alcohol, ml/d (mean)	5.3 ± 11.7	5.8 ± 10.9	6.0 ± 12.7	4.7 ± 10.0	0.483
Fibre, g/d (mean)	28.2 ± 13.9	26.6 ± 12.2	25.8 ± 11.8	23.6 ± 10.3	<0.001
**Daily food pyramid shelf servings**					
Bread, cereal, potatoes, grains and rice (mean)	5.1 ± 3.0	5.2 ± 2.7	5.5 ± 3.3	5.4 ± 2.9	0.026
Fruit and vegetables (mean)	9.2 ± 6.9	7.7 ± 4.9	6.5 ± 4.0	5.2 ± 3.1	<0.001
Dairy (mean)	1.7 ± 1.4	1.9 ± 1.4	2.1 ± 1.5	2.0 ± 1.6	<0.001
Meat, fish, poultry and eggs (mean)	2.3 ± 1.3	2.4 ± 1.2	2.5 ± 1.3	2.4 ± 1.4	0.026
Fats, high fat/sugar foods and drinks (mean)	4.6 ± 2.9	7.1 ± 4.0	8.4 ± 4.5	11.5 ± 5.7	<0.001

Abbreviations: FSAm-NPS: Food Standards Agency nutrient profiling system; MUFA: monounsaturated fatty acids; PUFA: polyunsaturated fatty acids; SFA: saturated fatty acids. *p* for trend determined from an ANOVA.

**Table 3:** Spearman correlation coefficients between the FSAm-NPS dietary index and inflammatory and thrombotic biomarkers.

Biomarker	Correlation Coefficients	*p*
C3, mg/dL	0.047	0.039
CRP, ng/mL	0.060	0.008
IL-6, pg/mL	0.074	0.001
TNF-α, pg/mL	0.078	0.001
Adiponectin, ng/mL	−0.115	<0.001
Leptin, ng/mL	−0.037	0.099
Resistin, ng/mL	0.032	0.162
PAI-1, ng/mL	0.039	0.086
WBC, 10^9^/L	0.104	<0.001
Neutrophils, 10⁹/L	0.116	<0.001
Lymphocytes, 10⁹/L	0.014	0.525
NLR	0.088	<0.001
Monocytes, 10⁹/L	0.107	<0.001
Eosinophils, 10⁹/L	0.069	0.002
Basophils, 10⁹/L	0.036	0.115

Values are presented as Spearman correlation coefficients between the FSAm-NPS dietary index and inflammatory and thrombotic biomarkers among the Mitchelstown cohort (n = 2006).

**Table 4:** Linear regression analysis of the associations between the FSAm-NPS dietary index and inflammatory and thrombotic biomarkers (n = 2006).

Biomarker	Model 1			Model 2			Model 3			Model 4		
	β	S.E.	*p*	β	S.E.	*p*	β	S.E.	*p*	β	S.E.	*p*
C3	1.330	0.558	**0.017**	1.654	0.564	**0.003**	1.666	0.591	**0.005**	1.767	0.571	**0.002**
Log CRP	0.045	0.016	**0.004**	0.055	0.016	**0.001**	0.048	0.017	**0.004**	0.047	0.016	**0.004**
Log IL-6	0.051	0.017	**0.003**	0.045	0.017	**0.007**	0.043	0.018	**0.013**	0.042	0.017	**0.016**
Log TNF-α	0.023	0.008	**0.004**	0.021	0.008	**0.008**	0.021	0.009	**0.012**	0.023	0.009	**0.009**
Log Adiponectin	−0.083	0.016	**<0.001**	−0.026	0.014	0.06	−0.025	0.015	0.082	−0.025	0.015	0.081
Log Leptin	−0.026	0.021	0.215	0.013	0.020	0.532	0.015	0.021	0.476	0.017	0.018	0.354
Log Resistin	0.020	0.010	**0.045**	0.026	0.010	**0.01**	0.027	0.010	**0.008**	0.029	0.010	**0.005**
PAI-1	0.417	0.284	0.142	0.158	0.286	0.582	0.089	0.302	0.768	0.055	0.307	0.857
Log WBC	0.028	0.006	**<0.001**	0.022	0.006	**0.001**	0.017	0.007	**0.007**	0.016	0.006	**0.01**
Log Neutrophils	0.037	0.008	**<0.001**	0.030	0.008	**<0.001**	0.025	0.008	**0.002**	0.024	0.008	**0.002**
Log Lymphocytes	0.006	0.007	0.421	0.006	0.007	0.462	0.002	0.008	0.757	0.002	0.008	0.775
Log NLR	0.031	0.009	**<0.001**	0.024	0.009	**0.006**	0.023	0.009	**0.016**	0.021	0.010	**0.028**
Log Monocytes	0.038	0.007	**<0.001**	0.024	0.007	**<0.001**	0.017	0.008	**0.021**	0.014	0.007	0.067
Log Eosinophils	0.046	0.014	**0.001**	0.033	0.014	**0.017**	0.035	0.015	**0.019**	0.037	0.015	**0.015**
Log Basophils	0.018	0.013	0.163	0.019	0.013	0.145	0.019	0.014	0.163	0.013	0.014	0.341

Model 1: univariate. Model 2: adjusted for age and sex. Model 3: adjusted for age, sex, anti-inflammatory medication use and physical activity. Model 4: adjusted for age, sex, anti-inflammatory medication use, physical activity, education, smoking, type 2 diabetes and BMI. Unstandardised β coefficients and standard errors (S.E.) are shown. Significant *p* in **bold**.

## Data Availability

The data used and analysed for the purpose of this study are available from the corresponding author on reasonable request.

## Supplementary Materials

The following supporting information can be downloaded at: https://www.mdpi.com/article/10.3390/nu14153122/s1, Supplementary File S1: FSAm-NPS, Nutrient Profiling System of the British Food Standards Agency.
