# Supplementary material for: Associations between the Nutrient Profiling System Underlying the Nutri-Score Nutrition Label and Biomarkers of Chronic Low-Grade Inflammation: A Cross-Sectional Analysis of a Middle- to Older-Aged Population
Source: Nutrients. 2022 Jul 29;14(15):3122. doi: 10.3390/nu14153122 (PMC9370507; doi:10.3390/nu14153122)
Supplement: Supplementary file 1 [file nutrients-14-03122-s001.zip › nutrients-1834138-supplementary.pdf]

Supplementary File S1. FSAm-NPS, Nutrient Profiling System of the British Food Standards Agency.

Points are allocated according to the nutrient content for 100g of foods or beverages. Points are allocated for 'Negative' nutrients (A points) and can be balanced according to 'Positive' nutrients (C points).

A points
Total A points = (points for energy) + (points for saturated fat) + (points for total sugar) + (points for sodium)

Points	**Energy (kJ)**	**Saturated Fat (g)**	**Total Sugars (g)**	**Sodium (mg)**
0	≤ 335	≤ 1	≤ 4.5	≤ 90
1	>335	>1	>4.5	>90
2	>670	>2	>9	>180
3	>1005	>3	>13.5	>270
4	>1340	>4	>18	>360
5	>1675	>5	>22.5	>450
6	>2010	>6	>27	>540
7	>2345	>7	>31	>630
8	>2680	>8	>36	>720
9	>3015	>9	>40	>810
10	>3350	>10	>45	>900

C points
Total C points = (points for fruits and vegetables) + (points for fibers) + (points for proteins)

Points	**Fruits/Vegetables/legumes/nuts (%)**	**Fiber (g) ***	**Protein (g)**
0	≤ 40	≤ 0.7	≤ 1.6
1	>40	>0.7	>1.6
2	>60	>1.4	>3.2
3	-	>2.1	>4.8
4	-	>2.8	>6.4
5	>80	>3.5	>8.0

*FSA score allocates different thresholds for fibers, depending on the measurement method used. We used NSP cut-offs to compute fibers score.

For 100g of a given food, the percentage of fruits/vegetables/legumes/nuts is obtained by summing up the amount (in grams) of all fruits, legumes and vegetables (including oleaginous fruits, dried fruits and olives) contained in this food.

Overall score computation

- If Total A points <11, then FSAm-NPS score =Total A points – Total C points
- If Total A points ≥11,

- If points for fruits/vegetables/legumes/nuts =5, then FSAm-NPS score =Total A points – Total C points
- Else if points for fruits/vegetables/legumes/nuts <5, then FSAm-NPS score = Total A points – (points for fiber + points for fruits/vegetables/legumes/nuts).

Exceptions were made for cheese, fat, and drinks to better rank them according to their nutrient profile, consistently with nutritional recommendations:

Score computation for cheese

For cheese, the score takes in account the protein content, whether the A score reaches 11 or not, i.e.: FSAm-NPS score =Total A points – Total C points

Score computation for added fat

For added fats (vegetable oils, margarines, butter), the criteria for point attribution is based on the percentage of saturated fat among total lipids instead of content of saturated fat, as shown below:

Points	**Saturated Fat/total lipids (%)**
0	<10
1	<16
2	<22
3	<28
4	<34
5	<40
6	<46
7	<52
8	<58
9	<64
10	≥64

Points attribution for the other nutrients follows the grid displayed in “A points” and “C points” above.

Score computation for beverages

For drinks, the grids for point attribution regarding energy, sugars and fruits/vegetables/ legumes/nuts (%) were modified.

Points	**Energy (kJ)**	**Total Sugar (g)**	**Fruits/Vegetables/legumes/nuts (%)**
0	≤0	≤0	<40
1	≤30	≤1.5	
2	≤60	≤3	>40
3	≤90	≤4.5	
4	≤120	≤6	>60
5	≤150	≤7.5	
6	≤180	≤9	
7	≤210	≤10.5	
8	≤240	≤12	
9	≤270	≤13.5	
10	Above	Above	>8

Points attribution for the other nutrients follows the grid displayed in “A points” and “C points” above.

Given the modification of the grid for fruit and vegetables for beverages, the threshold in the final computation to take into account protein content is set at 10 points:

- If Total A points <11, then FSAm-NPS score =Total A points – Total C points
- If Total A points ≥11,
    - If points for fruits/vegetables/legumes/nuts =10, then FSAm-NPS score =Total A points – Total C points
    - Else if points for fruits/vegetables/legumes/nuts <10, then FSAm-NPS score = Total A points – (points for fiber + points for fruits/vegetables/legumes/nuts).

Milk and vegetable milk are not concerned by this exception. Their scores are computed using the overall score computation system.
